# ZPR1 Is Dispensable for HPV R-Loop Resolution but Regulates Host R-Loop Dynamics

**DOI:** 10.3390/v17111502

**Published:** 2025-11-13

**Authors:** Rylann Moffitt, Steven Brooks, Elliot J. Androphy, Marsha DeSmet

**Affiliations:** 1 Tom and Julie Wood College of Osteopathic Medicine, Marian University, Indianapolis, IN 46222, USA; rmoffitt364@marian.edu; 2 Department of Medical and Molecular Genetics, Indiana University School of Medicine, Indianapolis, IN 46037, USA; stevbroo@iu.edu; 3 Department of Dermatology, Indiana University School of Medicine, Indianapolis, IN 46037, USA; eandro@iu.edu

**Keywords:** HPV transcription, SETX, ZPR1, R-loops, E2

## Abstract

The human papillomavirus (HPV) is a small, non-enveloped virus with a circular double-stranded DNA genome. The HPV genome encodes the E2 activator protein, which is required for viral transcription. R-loops are triple-stranded nucleic acid structures that occur when newly synthesized single-stranded RNA anneals to duplex DNA. These structures form during papillomavirus transcription. We and others have demonstrated that resolution of viral R loops is crucial for HPV episomal maintenance. ZPR1 is a zinc finger protein that can recruit SETX to mammalian R-loops to mediate resolution. E2 binds to and recruits SETX, an R-loop helicase, to the viral promoter. We observed E2 in complex with SETX and ZPR1. However, we found that ZPR1 depletion decreased viral R-loops while enhancing cellular R-loops. ZPR1 depletion also increased SETX binding to the viral promoter. These data suggest that ZPR1 is not required for HPV R-loop resolution, in contrast to what has been observed in mammalian cells. We detected the E2 protein associated with R-loops and found that E2 overexpression increases cell-derived R-loop formation. Analysis of TCGA datasets revealed that ZPR1, but not SETX, mRNA expression is significantly reduced in HPV-positive cervical and head and neck cancers. Together, these findings indicate that while E2 mediates HPV R-loop resolution, it also promotes R-loop accumulation in the host genome, likely through SETX sequestration.

## 1. Introduction

R-loops are triple-stranded nucleic acid structures that occur when newly synthesized single-stranded RNA anneals to duplex DNA and upon collision of replication forks with transcription complexes [1,2]. These hybrid structures form on the HPV [3,4,5] and other DNA tumor viruses genomes [6,7,8,9]. Human Senataxin (SETX) and its yeast homolog Sen1 were the first described RNA-DNA helicases involved in R-loop resolution [10,11]. Senataxin and Sen1 associate with replication forks and facilitate their movement through RNA polymerase II (RNAPII) transcription bubbles [12]. SETX also interacts directly with RNAPII and resolves R-loops during fork repair [13,14]. HPV R-loops resolution is dependent on SETX. This helicase associates with the HPV promoter in an E2-dependent manner and is required for transcriptional activation. Depletion of SETX blocks transcription and increases viral integration in cell lines that maintain viral episomes [3].

HPV replication and transcription are controlled by the viral E2 protein through the recognition and binding to E2 binding sites at the viral long-controlled region. The N-terminal 200 amino acids of the E2 transactivation domain provide a platform for protein–protein interactions [15]. The mechanism by which E2 recruits SETX, and the cellular conditions that govern this recruitment, remain undefined.

Zinc-finger protein ZPR1 is a nuclear and cytoplasmic protein demonstrated to bind the COOH-terminal domain (CTD) of RNA pol II [16]. ZPR1 binds and recruits SETX to DNA-RNA hybrids for resolution [17]. ZPR1 has been identified as a key factor in R-loop resolution in the context of spinal muscular atrophy (SMA) and other neurological disorders [10,17,18,19]. SMA is a lethal neuro-muscular disease caused by the mutation or deletion of the SMN1 gene. SMN binds to ZPR1, which modulates expression of the DNA-RNA helicase SETX [16]. Low levels of the SMN protein, which are the cause of SMA, lead to the accumulation of R-loops and double-stranded breaks [20], p53 activation [21], and the death of motor neurons [22]. Given its ability to recruit SETX to R-loops, we hypothesize that ZPR1 is a critical mediator of E2-dependent SETX recruitment and R-loop resolution on the HPV genome. Although ZPR1 has been implicated in R-loop resolution during mammalian transcription, its precise role in coordinating SETX activity with viral or cellular transcriptional machinery remains poorly defined. In this study, we investigated whether ZPR1 functions as a bridge between E2 and SETX at the HPV promoter and its effect on viral transcription and genome stability.

## 2. Materials and Methods

### 2.1. Plasmids and Antibodies

The plasmids used in this study were pCDNA3-FLAG-HPV31 E2, pCG-BPV-E2, BPV-LCR, and GFP-SETX (a gift from L. Gangwani). The antibodies included mouse anti-ZPR1 (LG-1, sc-3981), mouse anti-S9.6 (MABE1095), mouse anti-FLAG M2 (Sigma; St. Louis, MO, USA), anti-β-actin (Sigma), rabbit anti-hSETX (Novus Biologicals; Centennial, CO, USA; used for Western blotting and ChIP assays), and rabbit anti-HPV31 E2 (used for immunoprecipitation of HPV31 E2 in ChIP assays).

### 2.2. Cell Culture

All cell lines were maintained at 37 °C in a humidified atmosphere containing 5% CO_2_. HEK293TT cells (from J. Schiller and C. Buck) were cultured in Dulbecco’s modified Eagle’s medium (DMEM; Life Technologies; Carlsbad, CA, USA) supplemented with 10% fetal bovine serum (FBS; Peak Serum; Bradenton, FL, USA) and 100 U/mL penicillin/streptomycin (Life Technologies). CIN612 cells (from L. Laimins) were maintained in E medium with mitomycin C-treated J23T3 fibroblast feeder cells (from H. Green).

### 2.3. DNA-RNA Co-Immunoprecipitation (DRIP)

Genomic DNA was isolated using the Qiagen (Hilden, Germany) Blood and Tissue Extraction Kit and digested overnight at 37 °C with a combination of restriction enzymes (HindIII, BsrGI, XbaI, NspI, and SspI). For hybrid immunoprecipitation, 15–20 µg of digested DNA was incubated with S9.6 antibody in 1× binding buffer (10 mM NaPO_4_ pH 7.0, 140 mM NaCl, 0.05% Triton X-100) overnight at 4 °C. DNA–antibody complexes were then captured with protein A/G magnetic beads (Active Motif; Carlsbad, CA, USA) for 2 h at 4 °C. Beads were washed three times with 1× binding buffer, and bound DNA was eluted in 200 µL of elution buffer (50 mM Tris-HCl pH 8.0, 10 mM EDTA, 0.5% SDS) containing proteinase K, followed by incubation for 45 min at 55 °C. DNA was isolated using a DNaeasy Kit (Qiagen). For qPCR analysis, the following primer pairs were used: 5′-AAAGTGGTGAACCGAAAACG-3′ and 5′-CATGCAATTTCCGAGGTCTT-3′. qPCR was completed using a BioRad (Hercules, CA, USA) CFX96 real-time PCR machine with CFX Manager 3.1.

### 2.4. Viral Transcription Analysis

CIN612 were transfected with Lipofectamine 2000 (Invitrogen) and either control siRNA A or C (Santa Cruz Biotechnology; Dallas, TX, USA; sc-37007, sc-44231) or ZPR-1 siRNA (sc-35282) at a final concentration of 15 nM in E medium. At 48 h post-transfection, total RNA was extracted using TRIzol (Invitrogen; Cambridge, MA, USA) and reverse transcribed into cDNA using the BioRad iScript cDNA Synthesis Kit). Quantitative real-time PCR was performed with SYBR Green (Bio-Rad). HPV31 E1^E4, HPV31 E8^E2, HPV31 E6, β-actin transcripts were quantified using previously described primers [23].

### 2.5. Chromatin Immunoprecipitation (ChIP) Assay

ChIP assays were performed using the ChIP-IT Express kit (Active Motif) according to the manufacturer’s protocol. Real-time PCR was carried out using SsoFast EvaGreen Supermix (Bio-Rad). The following primer pairs were used: HPV31 P97, 5′-AAAGTGGTGAACCGAAAACG-3′ and 5′-CATGCAATTTCCGAGGTCTT-3′. Data was analyzed using Bio-Rad CFX Manager 3.1 software.

### 2.6. Co-Immunoprecipitation (Co-IP)

Cells were transfected with polyethylenimine (PEI; 2 mg/mL) and harvested 48 h later. Cells were lysed in buffer containing 0.5% NP-40, 300 mM NaCl, 20 mM HEPES (pH 7.4), 1 mM pervanadate, Benzonase nuclease, and protease inhibitor cocktail (Sigma, St. Louis, MO, USA). For each reaction, ~1 µg of antibody and 30 µL of a 50% protein A/G bead slurry (Invitrogen) were added and rotated overnight at 4 °C. Beads were washed three times with lysis buffer, and bound proteins were resolved by SDS–polyacrylamide gel electrophoresis (SDS–PAGE). Proteins were transferred to polyvinylidene difluoride (PVDF) membranes (Millipore; Burlington, MA, USA), blocked with 5% TBS–Tween–milk, and probed with the indicated antibodies. Signals were detected using chemiluminescent substrates (Thermo Scientific, Cambridge, MA, USA). Inputs represent 10% of the starting lysate. For DRIP-immunoblot experiments, cells were lysed in the buffer described above and sonicated for 10 s on ice. Lysates were spun at 15,000 rpm for 10 min and subjected to nuclease digestion at 37 °C for 1 h. Lysates were then placed with magnetic beads and S9.6 antibody for rotation at 4 °C 1 overnight. Immunocomplexes were washed and subjected to immunoblot as described above.

### 2.7. Exonuclease V Digestion

Cell pellets were resuspended in PBS pH 7.4, and genomic DNA was isolated using the DNeasy Blood and Tissue Kit (Qiagen). A total of 500 ng of DNA was either treated with Exonuclease V (New England Biolabs, Ipswich, MA, USA) or left untreated for 90 min at 37 °C. The enzyme was inactivated at 65 °C for 20 min. For quantification, 50 ng of digested or undigested DNA was analyzed by real-time PCR using previously described HPV31 and actin primers. The percentage of Exonuclease V-resistant DNA was calculated using the formula:

% resistant DNA = 2^−(Cᴛ digested−Cᴛ undigested)^ × 100.

### 2.8. SETX and ZPR1 Expression Analysis

Data sources and cohort definition: We analyzed RNA-seq data from The Cancer Genome Atlas (TCGA) for cervical squamous cell carcinoma and endocervical adenocarcinoma (TCGA-CESC) and head and neck squamous cell carcinoma (TCGA-HNSC). Gene Expression Quantification files generated with the “STAR-Counts” workflow were retrieved from the NCI Genomic Data Commons (GDC) via TCGAbiolinks 2.32.0 and prepared into SummarizedExperiment 1.34.0 objects using GDCprepare. For HNSC, only Primary Tumor samples were requested.

For HNSC, the primary HPV variable was derived from TCGA clinical data: patients were classified HPV-positive if either p16 IHC or HPV ISH was positive, HPV-negative if both were negative, and otherwise set to missing. For CESC, HPV status per barcode was taken from the curated Evans et al. resource from the supplement. restricted to high-risk HPV types (16, 18, 31, 33, 35, 39, 45, 51, 52, 56, 58, 59). Clinical calls reflect the contemporaneous diagnostic assays used in patient care and remain a common reference in TCGA studies; therefore, these were prespecified as our primary labels. To address label-definition concerns, we repeated the analysis using RNA-seq–derived HPV calls available for TCGA [24].

Gene expression quantification and transformation: Prepared SummarizedExperiment objects contained an unstranded TPM assay used for downstream analyses. For each gene of interest, TPM values were transformed as log (TPM + 1). We focused on SETX (ENSG00000107290) and ZPR1 (ENSG00000109917). Ensembl gene rows were located by matching the stable ID prefix (anchored to the ID with a version suffix).

Statistical analysis: Within each cancer type, differences in log-transformed expression between HPV-negative and HPV-positive groups were assessed using a two-sided Welch’s *t*-test (unequal variances), conducted separately for SETX and ZPR1.

Visualization: For each gene and cohort, we plotted violin distributions overlaid with quasirandom point clouds (to display all observations), marked group medians, and annotated the Welch test *p*-value.

Software: all analyses were performed in R using TCGAbiolinks [25], SummarizedExperiment [26], dplyr 1.1.4 [27], ggplot2 3.5.2 [28], ggbeeswarm 0.7.2, patchwork 1.2.0, cowplot 1.1.3, and ragg 1.3.2.

The source code for the analysis can be accessed at: https://github.com/stevenb21/ZPR1_TCGA (7 September 2025) The repository includes scripts to (i) pull data from GDC, (ii) annotate HPV status (CESC via Evans et al. supplemental table; HNSC via clinical p16/ISH), (iii) build patient-level SummarizedExperiment objects, and (iv) regenerate publication figures. Mapping tables linking RNA-seq columns, patient IDs, and HPV status are written to disk for auditing.

### 2.9. Statistical Analysis

Cell culture data were analyzed using one-way or two-way Student’s *t* tests as appropriate. Values are presented as mean ± standard error of the mean (SEM). *p* values ≤ 0.05 were considered statistically significant.

### 2.10. Data Availability

The data supporting the findings of this study are available from the corresponding author upon reasonable request.

## 3. Results

### 3.1. ZPR1 Is in Complex with SETX-E2

**Figure 1:**
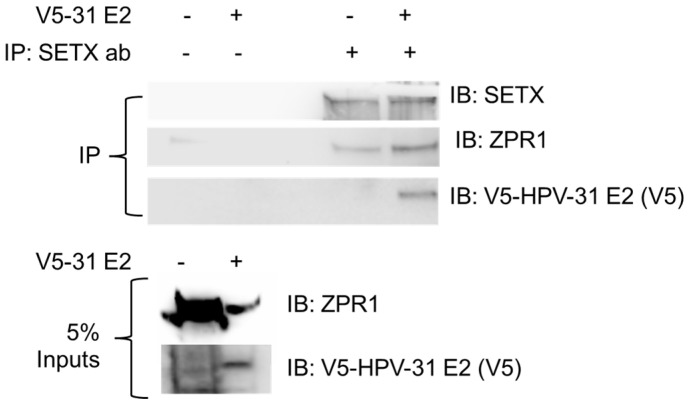
HPV-E2 is in complex with ZPR-1 and SETX. HEK293TT cells were transfected with GFP-SETX and FLAG-ZPR1 with and without FLAG-HPV-31E2 constructs. SETX was immunoprecipitated with SETX antibodies. Immunocomplexes were blotted with SETX, ZPR1, and V5 (HPV-31 E2) antibodies.

We previously identified the HPV E2 protein in complex with the RNA–DNA helicase SETX [3]. ZPR1 is known to bind R-loops and SETX to recruit this helicase to these structures [17], although much of this work has been described in the context of neurological disease. Our earlier studies also suggested that E2 may facilitate SETX association at the viral promoter [3]. Therefore, we first asked whether ZPR1 could form a complex with E2 and SETX, hypothesizing that it may serve as a participant in this pathway. To test this, HEK293TT cells were transfected with V5-tagged HPV-31 E2, SETX, and FLAG-ZPR1 constructs. As shown in Figure 1, SETX co-immunoprecipitated with ZPR1, and consistently, E2 was also detected within this complex. This data suggested that ZPR1 may contribute to E2-mediated R-loop resolution and or transcription.

### 3.2. ZPR1 Depletion Decreases HPV R-Loops and Increases SETX Loading to the Viral Promoter

**Figure 2:**
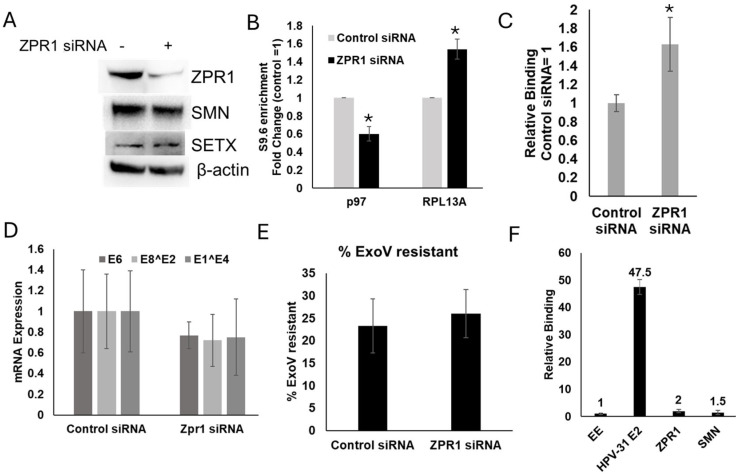
Viral R-loops are decreased with ZPR1 depletion. Episomal CIN612 cells were treated with ZPR1 siRNAs for 48 hrs. (**A**) ZPR1 depletion did not change SMN or SETX protein levels. (**B**) DNA was isolated, and R-loop complexes were immunoprecipitated with S9.6 antibodies/Protein G beads. DNA was eluted from S9.6 antibodies, and quantitative PCR was performed using primers flanking the P97 promoter or a known mammalian R-loops region (RPL13A) and normalized to input DNA. Mean ± SEM, *n* = 6, * *p* < 0.05 by 2-way *t*-test. (**C**) ChIP assays were performed on lysates ± ZPR1 siRNA using SETX antibodies and primers flanking the HPV p97 promoter. Mean ± SEM, *n* = 7, * *p* < 0.05 by 2-way *t*-test. Control was set to 1. (**D**) RNA was isolated from episomal CIN612 cells ± ZPR1 siRNA, and E6, E8^E2, and E1^E4 expression was normalized to β-actin mRNA. Mean ± SEM, *n* = 3–4. (**E**) CIN612 cells were treated with control or ZPR1 siRNA for 96 h. DNA was isolated and subjected to Exonuclease V digestion. Values are expressed as means ± SEM, *n* = 8. (**F**) ChIP assays were performed on CIN612 lysates with primers to the viral promoter. Antibodies tested were HPV-31 E2, ZPR1, and SMN. EE was used as a non-specific IgG control, and fold change to EE is written at the top of each bar. Mean ± SEM, *n* = 3.

We predicted that if ZPR1 were necessary for R-loop resolution, its depletion would lead to an increase in HPV R-loops. To test this, HPV-31 episomal cervical CIN612 cells were treated with ZPR1 siRNA for 48 h. ZPR1 knockdown did not affect SETX or SMN protein levels, in contrast to what has been reported in SMA disease cell lines (Figure 2A). DNA was then isolated and subjected to DNA–RNA immunoprecipitation (DRIP)-qPCR using primers specific to the HPV p97 promoter. Surprisingly, ZPR1 depletion significantly decreased R-loops at this region (Figure 2B), contrary to our hypothesis. Consistent with this finding, SETX binding to the p97 promoter was significantly increased following ZPR1 depletion (Figure 2C). This result is consistent with the idea that increased SETX recruitment was responsible for the suppression of R-loops. However, ZPR1 depletion significantly increased R-loops at a known R-loop focus in the host genome (Figure 2B). We did not detect changes in viral transcription (Figure 2D) or episomal maintenance (Figure 2E) following ZPR1 depletion, since it did not increase R-loop formation and block transcription on the viral genome like we previously observed with SETX depletion [3]. We detected only modest binding of ZPR1 and SMN to the viral promoter in CIN612 cells (Figure 2F). Together, these data suggest that ZPR1 is necessary for R-loop resolution on the CIN612 host genome, as shown, but not on the HPV genome.

### 3.3. E2 Associates with RNA-DNA Hybrid-Containing Chromatin

**Figure 3:**
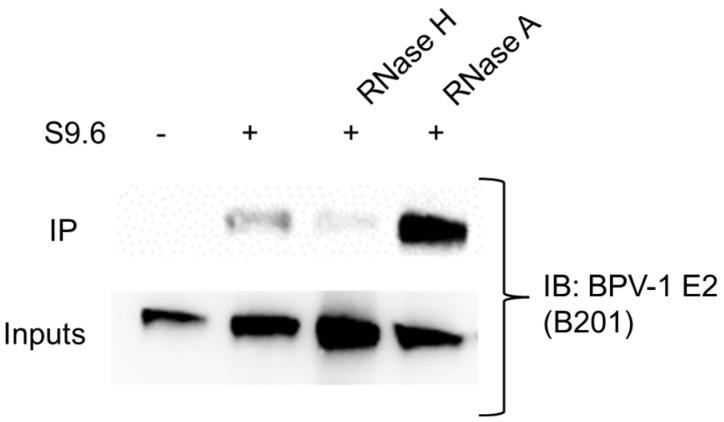
E2 binds to R-loops. HEK293TT cells were transfected with BPV-1 E2, GFP-SETX, and BPV-LCR constructs. Lysates were treated with RNase H or RNase A. R-loop complexes were immunoprecipitated with S9.6 antibodies/Protein G beads. Samples were subjected to immunoblot with BPV-1 E2 antibodies (B201).

R-loop binding proteins are critical regulators of R-loop dynamics. For example, ZPR1 binds to R-loops to facilitate SETX recruitment and activity of these structures [20]. Our data suggested that ZPR1 is not an important regulator of HPV transcription. Instead, we hypothesize that E2, rather than ZPR1, may facilitate SETX association. This hypothesis is based on our previous findings that SETX alone was not sufficient to stimulate E2-driven transcription from a reporter containing E2 binding sites [3], and on the observation that ZPR1 depletion did not increase viral R-loops. To further support this model, we asked whether E2 could bind to R-loops. Originally, we aimed to test this hypothesis using HPV-16 and HPV-31 E2 proteins; however, we were unable to achieve sufficient expression levels for this sensitive assay. To overcome this limitation, HEK293TT cells were transfected with BPV-1 E2, SETX, and BPV-LCR constructs. We chose BPV-1 E2 because it is expressed at high levels, and we have well-validated antibodies that reliably detect this protein under sensitive conditions. Lysates were subjected to the following immunoprecipitation conditions: (1) no S9.6 antibody (negative control for R-loop detection), (2) S9.6 antibody, (3) S9.6 antibody plus RNase H treatment, and (4) S9.6 antibody plus RNase A treatment. RNase H was included as a positive control for R-loop specificity, as it cleaves the RNA strand of RNA:DNA hybrids, whereas RNase A served as an enzymatic control that should not degrade R-loops. DNA-hybrid complexes were blotted with BPV-1 E2 antibodies. As shown in Figure 3, E2 was enriched in the S9.6 antibody and the S9.6 antibody plus RNase A treatment groups. These data support a model in which E2 contributes to R-loop regulation by engaging hybrid-containing chromatin complexes that may include SETX.

### 3.4. E2 Overexpression Increases Host R-Loop Formation

**Figure 4:**
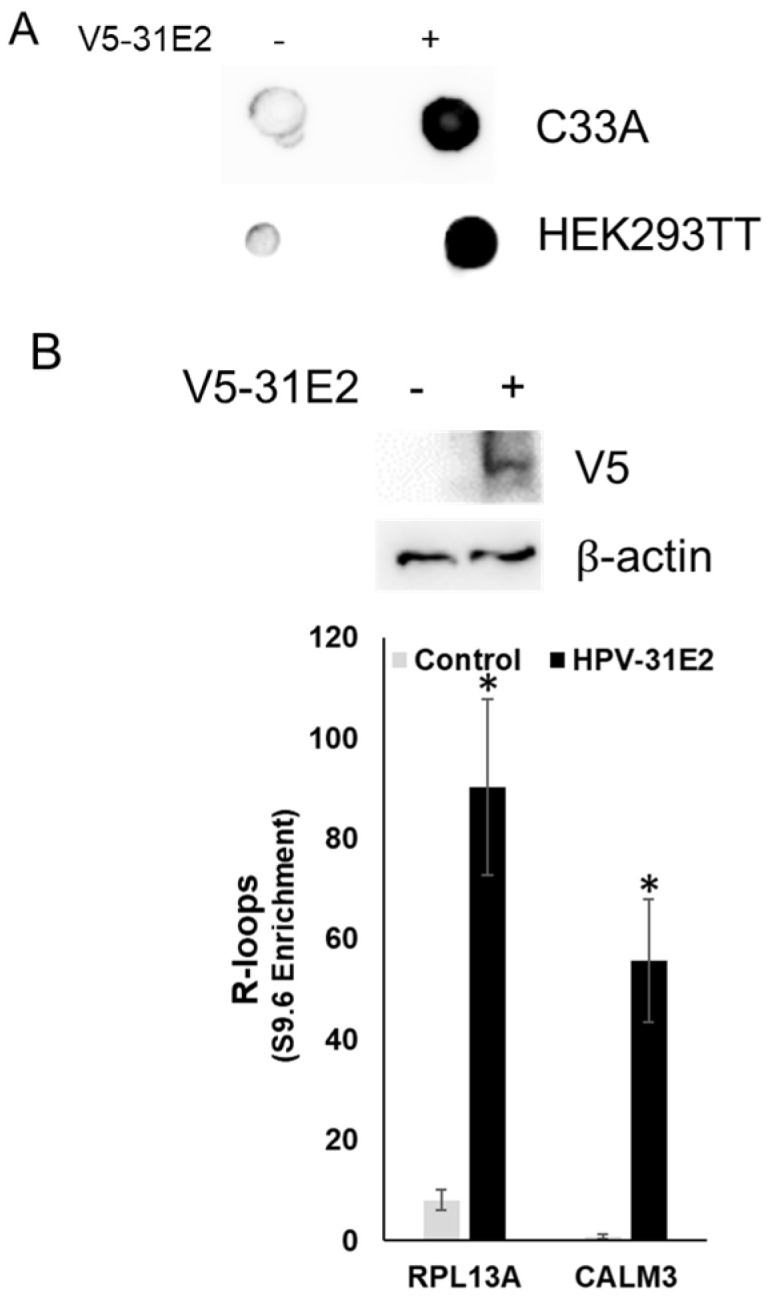
R-loops are increased in cell lines following HPV E2 overexpression. (**A**) C33A and HEK293TT cells were transfected with HPV-31 E2. Forty-eight hours later, DNA was quantified, isolated, and blotted onto a nylon membrane. Blot was immunoblotted with S9.6 (detects R-loops) antibodies. (**B**) C33A cells were transfected with and without HPV-31E2 constructs. DNA was isolated, and R-loop complexes were immunoprecipitated with S9.6 antibodies/Protein G beads. DNA was eluted from S9.6 antibodies, and quantitative PCR was performed using primers that detect known mammalian R-loop regions (RPLA13, CALM3) and normalized to input DNA. Mean ± SEM, *n* = 6, * *p* < 0.05 by 2-way *t*-test.

If E2, but not ZPR1, is required for SETX recruitment to viral R-loops and their resolution, we questioned why ZPR1 depletion suppressed viral R-loops while increasing mammalian R-loops. We hypothesize that elevated levels of E2 may compete SETX away from ZPR1, resulting in decreased R-loops at the viral genome but increased R-loops on the host genome. This hypothesis is supported by our data showing increased SETX association with the viral promoter upon ZPR1 depletion. To further test this model, C33A and HEK293TT cells were transfected with HPV-31 E2, and global R-loops were measured. E2 overexpression increased R-loops as detected by dot blot analysis with S9.6 antibodies (Figure 4A). Consistently, DRIP-PCR in C33A cells revealed elevated R-loops at two known host R-loop foci, RPL13A and CALM3 (Figure 4B).

### 3.5. ZPR1, but Not SETX Levels Are Lower in HPV Positive Cervical and Head And Neck Cancers

**Figure 5:**
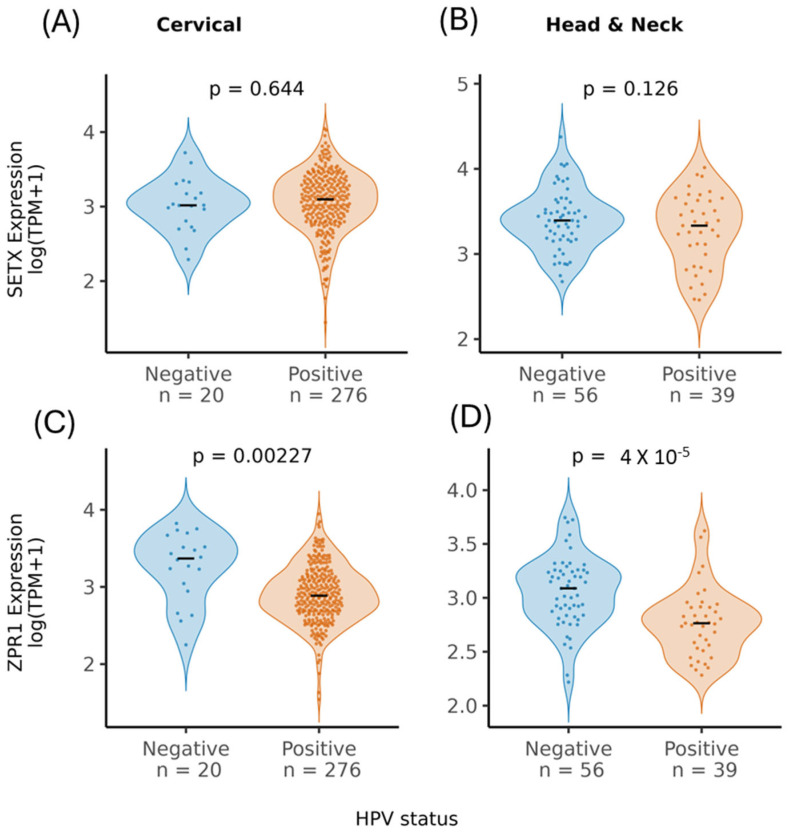
SETX and ZPR1 mRNA expression in HPV positive cervical and Head And Neck cancers. We analyzed RNA-seq data from The Cancer Genome Atlas (TCGA) for cervical squamous cell carcinoma and endocervical adenocarcinoma (TCGA-CESC) and head and neck squamous cell carcinoma (TCGA-HNSC). We restricted to high-risk HPV types (16, 18, 31, 33, 35, 39, 45, 51, 52, 56, 58, 59). SETX (**A**,**B**) and ZPR1 (**C**,**D**) mRNA expression was compared between HPV-negative and HPV-positive cancers.

We next examined SETX and ZPR1 expression in HPV-positive cervical and head and neck cancers using mRNA data from the TCGA database (Figure 5). Switching from clinical to RNA-seq HPV labels did not change the direction or interpretation of SETX and ZPR1 differences in either CESC or HNSC.SETX expression did not differ between HPV-negative and HPV-positive cancers (Figure 5A,B). In contrast, ZPR1 expression was significantly lower in HPV-positive cervical and head and neck cancers (Figure 5C,D). This observation is notable, as it suggests that lower ZPR1 expression in HPV-positive cancers might make ZPR1 more available for recruitment by E2, rather than tightly coupled to SETX; however, because E2 levels in these tumors are unclear and this finding is based on observational transcriptomic data, not proteomics, further studies will be required to determine functional significance.

## 4. Discussion

We initially hypothesized that ZPR1 would be necessary for viral R-loop resolution because we observed the protein in complex with both SETX and E2. ZPR1 associates with SETX independently of E2, and SETX has also been shown to interact with E2 [3]. However, it remains unclear whether these proteins form a single complex or whether we are capturing independent interactions. Resolving this question would require an in vitro system, which cannot replicate the cellular environment or account for post-translational modifications. In addition, our studies rely on high protein expression because these proteins are not easy to detect under endogenous conditions. As noted previously, our studies rely on high protein expression because SETX (~300 kDa) and ZPR1 are difficult to detect and efficiently isolate at endogenous levels. We therefore performed these assays in HEK293TT cells, which tolerate high E2 expression, unlike keratinocyte models, where these levels can be cytotoxic. As a result, we may not be capturing the authentic E2-SETX complex. For example, if E2 sequesters SETX away from ZPR1 and mammalian R-loop resolution, one might expect reduced interaction between ZPR1 and SETX. We did not observe this; however, such an effect may only be detectable when proteins are present at endogenous levels.

In neurological systems, ZPR1 has been shown to bind R-loops and recruit SETX for their resolution [20]. For example, reduced ZPR1 levels are associated with spinal muscular atrophy (SMA) and aberrant R-loop accumulation [16]. Consistent with this, we observed that low levels of ZPR1 increased mammalian R-loops in HPV-infected cell lines. However, in contrast, reduced ZPR1 enhanced SETX association with the viral promoter and suppressed R-loop formation on the HPV genome. These observations suggest that reduced ZPR1 levels may promote HPV transcription by facilitating R-loop resolution at the viral promoter.

This is one of the first studies to identify a HPV protein directly involved in the R-loop complex. We found that E2 overexpression altered mammalian R-loop resolution. Templeton et al. recently reported that HPV overexpression increases global R-loops [4], and that this effect was mediated through E6-dependent degradation of p53. Here, we present an additional mechanism for HPV-induced R-loops, mediated through the E2 protein. It remains unclear what levels of E2 are sufficient to induce R-loop formation, and whether this process is influenced by p53 status. During differentiation-dependent viral amplification, E2 expression is hypothesized to increase. This may represent a window in the viral life cycle where HPV influences host R-loop dynamics by drawing SETX to the viral origin, thereby supporting HPV transcription and replication.

In this study, we identified the viral E2 protein as a master regulator of R-loop resolution. E2 overexpression increased mammalian R-loops, which we predict is mediated through disruption of the SETX–ZPR1 interaction. We acknowledge that SETX may still associate with ZPR1; however, the SETX–E2 interaction may be favored during HPV infection, potentially altering SETX availability at host versus viral loci. Elevated R-loops create an environment of genomic instability and interfere with DNA repair pathways [1,29]. Such events may facilitate HPV in carrying out the productive transcriptional and replication stages of its life cycle. At the same time, HPV must avoid genome integration. Although integration events contribute to HPV-associated oncogenesis, they disrupt the viral life cycle by preventing episomal maintenance and the production of new virions. This is not surprising, as E2 must regulate the viral genome, and integration often disrupts its expression.

**Figure 6:**
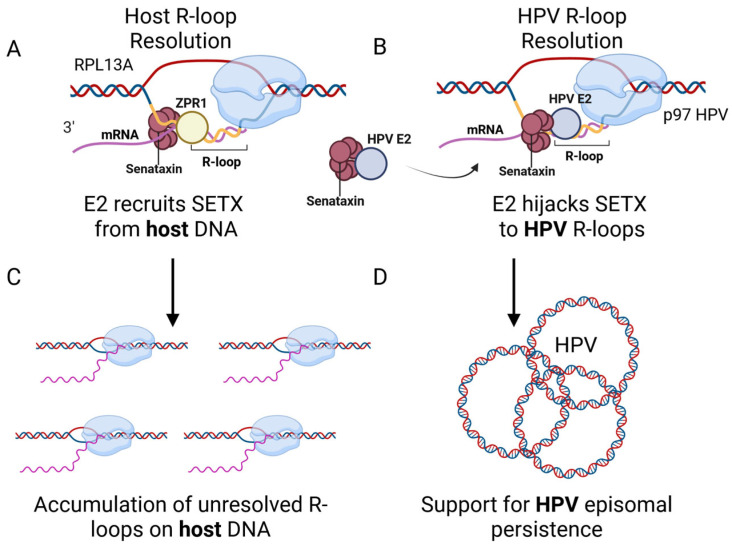
Proposed model for E2-mediated modulation of R-loop resolution during HPV episomal maintenance. (**A**) E2 may compete with ZPR1 for SETX association. (**B**) E2 associates with R-loop complexes to direct SETX to viral R-loops. (**C**) Reduced SETX on the host chromatin may cause increased R-loops. (**D**) SETX resolution of viral R-loops maintains HPV replication and transcription. Created in BioRender. DeSmet, 2025. https://BioRender.com.

We present the following model: during episomal maintenance through viral transcription and replication, R-loops form on the HPV episome. E2 may compete with ZPR1 for SETX binding to R-loops and recruiting SETX to these regions, including E2 binding sites in the early promoter (Figure 6). This redistribution could reduce SETX availability for host genome maintenance, leading to increased host R-loops (Figure 6C), while preserving R-loop resolution on the HPV episome to support viral transcription and replication (Figure 6D). Additional studies will determine whether E2 overexpression induces R-loops through p53, a known E2 binding partner [30], and whether this mechanism occurs during amplification of the viral genome. Together, these data provide strong evidence that the host protein ZPR1 is not required for viral R-loop resolution but remains important for regulating mammalian transcriptional dynamics. This study highlights how HPV exploits and disrupts host transcriptional processes to ensure completion of its life cycle.

## Data Availability

The data presented in this study are available upon request from the corresponding author.

## References

[1] Allison D.F., Wang G.G. (2019). R-loops: Formation, function, and relevance to cell stress. Cell Stress.

[2] Niehrs C., Luke B. (2020). Regulatory R-loops as facilitators of gene expression and genome stability. Nat. Rev. Mol. Cell Biol..

[3] Jose L., Smith K., Crowner A., Androphy E.J., DeSmet M. (2024). Senataxin mediates R-loop resolution on HPV episomes. J. Virol..

[4] Templeton C.W., Laimins L.A. (2023). p53-dependent R-loop formation and HPV pathogenesis. Proc. Natl. Acad. Sci. USA.

[5] Templeton C.W., Laimins L.A. (2024). HPV induced R-loop formation represses innate immune gene expression while activating DNA damage repair pathways. PLoS Pathog..

[6] Crowner A., Smith K., DeSmet M. (2024). Regulation of R-Loops in DNA Tumor Viruses. Pathogens.

[7] Wongsurawat T., Gupta A., Jenjaroenpun P., Owens S., Forrest J.C., Nookaew I. (2020). R-loop-forming Sequences Analysis in Thousands of Viral Genomes Identify A New Common Element in Herpesviruses. Sci. Rep..

[8] Rennekamp A.J., Lieberman P.M. (2011). Initiation of Epstein-Barr virus lytic replication requires transcription and the formation of a stable RNA-DNA hybrid molecule at OriLyt. J. Virol..

[9] Yiu S.P.T., Guo R., Zerbe C., Weekes M.P., Gewurz B.E. (2022). Epstein-Barr virus BNRF1 destabilizes SMC5/6 cohesin complexes to evade its restriction of replication compartments. Cell Rep..

[10] Skourti-Stathaki K., Proudfoot N.J., Gromak N. (2011). Human senataxin resolves RNA/DNA hybrids formed at transcriptional pause sites to promote Xrn2-dependent termination. Mol. Cell.

[11] Mischo H.E., Gómez-González B., Grzechnik P., Rondón A.G., Wei W., Steinmetz L., Aguilera A., Proudfoot N.J. (2011). Yeast Sen1 helicase protects the genome from transcription-associated instability. Mol. Cell.

[12] Alzu A., Bermejo R., Begnis M., Lucca C., Piccini D., Carotenuto W., Saponaro M., Brambati A., Cocito A., Foiani M. (2012). Senataxin Associates with Replication Forks to Protect Fork Integrity across RNA-Polymerase-II-Transcribed Genes. Cell.

[13] Brambati A., Zardoni L., Achar Y.J., Piccini D., Galanti L., Colosio A., Foiani M., Liberi G. (2018). Dormant origins and fork protection mechanisms rescue sister forks arrested by transcription. Nucleic Acids Res..

[14] Gatti V., De Domenico S., Melino G., Peschiaroli A. (2023). Senataxin and R-loops homeostasis: Multifaced implications in carcinogenesis. Cell Death Discov..

[15] Morgan I.M. (2025). The functions of papillomavirus E2 proteins. Virology.

[16] Kannan A., Jiang X., He L., Ahmad S., Gangwani L. (2020). ZPR1 prevents R-loop accumulation, upregulates SMN2 expression and rescues spinal muscular atrophy. Brain.

[17] Kannan A., Gangadharan Leela S., Branzei D., Gangwani L. (2024). Role of senataxin in R-loop-mediated neurodegeneration. Brain Commun..

[18] Kannan A., Cuartas J., Gangwani P., Branzei D., Gangwani L. (2022). Mutation in senataxin alters the mechanism of R-loop resolution in amyotrophic lateral sclerosis 4. Brain.

[19] Perego M.G.L., Taiana M., Bresolin N., Comi G.P., Corti S. (2019). R-Loops in Motor Neuron Diseases. Mol. Neurobiol..

[20] Cuartas J., Gangwani L. (2022). R-loop Mediated DNA Damage and Impaired DNA Repair in Spinal Muscular Atrophy. Front. Cell. Neurosci..

[21] Simon C.M., Dai Y., Van Alstyne M., Koutsioumpa C., Pagiazitis J.G., Chalif J.I., Wang X., Rabinowitz J.E., Henderson C.E., Pellizzoni L. (2017). Converging Mechanisms of p53 Activation Drive Motor Neuron Degeneration in Spinal Muscular Atrophy. Cell Rep..

[22] Kannan A., Bhatia K., Branzei D., Gangwani L. (2018). Combined deficiency of Senataxin and DNA-PKcs causes DNA damage accumulation and neurodegeneration in spinal muscular atrophy. Nucleic Acids Res..

[23] Jose L., Androphy E.J., DeSmet M. (2022). SETD6 Regulates E2-Dependent Human Papillomavirus Transcription. J. Virol..

[24] Evans A.M., Salnikov M., Tessier T.M., Mymryk J.S. (2022). Reduced MHC Class I and II Expression in HPV-Negative vs. HPV-Positive Cervical Cancers. Cells.

[25] Colaprico A., Silva T.C., Olsen C., Garofano L., Cava C., Garolini D., Sabedot T.S., Malta T.M., Pagnotta S.M., Castiglioni I. (2016). TCGAbiolinks: An R/Bioconductor package for integrative analysis of TCGA data. Nucleic Acids Res..

[26] Gopaulakrishnan S., Pollack S., Stubbs B.J., Pagès H., Readey J., Davis S., Waldron L., Morgan M., Carey V. (2019). restfulSE: A semantically rich interface for cloud-scale genomics with Bioconductor. F1000Research.

[27] Broatch J.E., Dietrich S., Goelman D. (2019). Introducing Data Science Techniques by Connecting Database Concepts and dplyr. J. Stat. Educ..

[28] Skidmore Z.L., Wagner A.H., Lesurf R., Campbell K.M., Kunisaki J., Griffith O.L., Griffith M. (2016). GenVisR: Genomic Visualizations in R. Bioinformatics.

[29] Li F., Zafar A., Luo L., Denning A.M., Gu J., Bennett A., Yuan F., Zhang Y. (2023). R-Loops in Genome Instability and Cancer. Cancers.

[30] Massimi P., Pim D., Bertoli C., Bouvard V., Banks L. (1999). Interaction between the HPV-16 E2 transcriptional activator and p53. Oncogene.

